# Oldest fossil remains of the enigmatic pig-footed bandicoot show rapid herbivorous evolution

**DOI:** 10.1098/rsos.160089

**Published:** 2016-08-10

**Authors:** Kenny J. Travouillon

**Affiliations:** Western Australian Museum, Welshpool, Western Australia 6106, Australia

**Keywords:** *Chaeropus*, Peramelemorphia, Fisherman's cliff, Pliocene, arid adaptation, new species

## Abstract

The pig-footed bandicoot, *Chaeropus ecaudatus,* is one of the most enigmatic Australian marsupials, which went extinct in the late 1950s probably as a result of European colonization. It is unusual in being the only marsupial to have evolved reduction of digits on both fore and hind feet, with the forefeet being pig-like (two toes) and the hind feet being horse-like (one toe). According to molecular phylogenetic analyses, *Chaeropus* diverged from other bandicoots (Peramelidae), and the bilbies (Thylacomyidae) by the mid-Late Oligocene. This is considerably earlier than suggested by the fossil record, with the current oldest specimens being Late Pleistocene in age. Here, I report the oldest fossils of *Chaeropus,* representing a new species, *Chaeropus baynesi* from the Late Pliocene to Early Pleistocene (2.47–2.92 Ma) Fisherman's Cliff Local Fauna, Moorna Formation, New South Wales, Australia, and extending the fossil record of the genus and family by at least 2 million years. *Chaeropus baynesi* is less high crowned than *C. ecaudatus* and lacks lateral blade development on lower molars, suggesting that it was unlikely to be grazing. This suggests that *Chaeropus* must have adapted rapidly to the drying conditions and changes in environments, and would have become a grazer in a very short period of time.

## 1. Introduction

**Figure 1.**
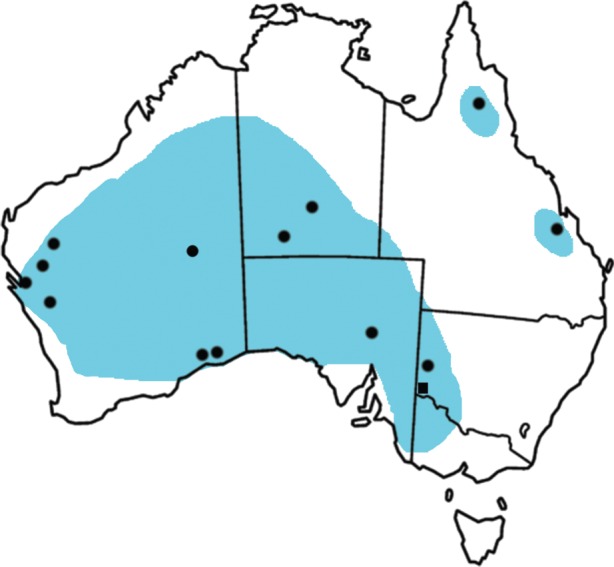
Distribution map of the pig-footed bandicoot, *Chaeropus ecaudatus,* prior to its extinction. Black dots represent fossil localities where it was found. Black square shows the location of the Moorna Formation.

The pig-footed bandicoot, *Chaeropus ecaudatus,* is one of the 30 species of mammals that went extinct in Australia after European settlement [1]. It went extinct sometime in the late 1950s, probably as a result of competition and predation by introduced species, and change in fire regime [2]. It is the only marsupial to walk on reduced digits both on the fore and hind feet [3], and unlike traditional bandicoots, which are insectivorous or omnivorous, *C. ecaudatus* was primarily a grazer with high-crown teeth [4]. Three species of *Chaeropus* were first described, *C. ecaudatus* [5], *Chaeropus castanotis* [6] and *Chaeropus occidentalis* [7], but only *C. ecaudatus* is valid today [8]. As the sole member of the family Chaeropodidae, it is most closely related to the bilbies (*Macrotis*) and diverged sometime in the mid-Late Oligocene, according to a dated molecular phylogeny [9]. In the fossil record, *C. ecaudatus* has been recovered from various sites across Australia dating from Late Pleistocene to Holocene (figure 1). The specimens described here represent a new species of *Chaeropus* and the oldest fossil record of the genus. The specimens were collected by Marshall [10] from the Moorna Formation (Fisherman's Cliff Local Fauna) in the southwest of New South Wales, Australia, magnetostratigraphically dated to 2.47–2.92 Ma [11].

High systematic nomenclature follows Jackson & Groves [8]. Dental nomenclature follows Travouillon *et al.* [12]. Specimens are registered with Museum Victoria, Melbourne, Australia.

## 2. Systematic palaeontology

*Infraclass.* Metatheria

*Order.* Peramelemorphia

*Family.* Chaeropodidae

Genus. Chaeropus

*Species. baynesi* new

**Figure 2.**
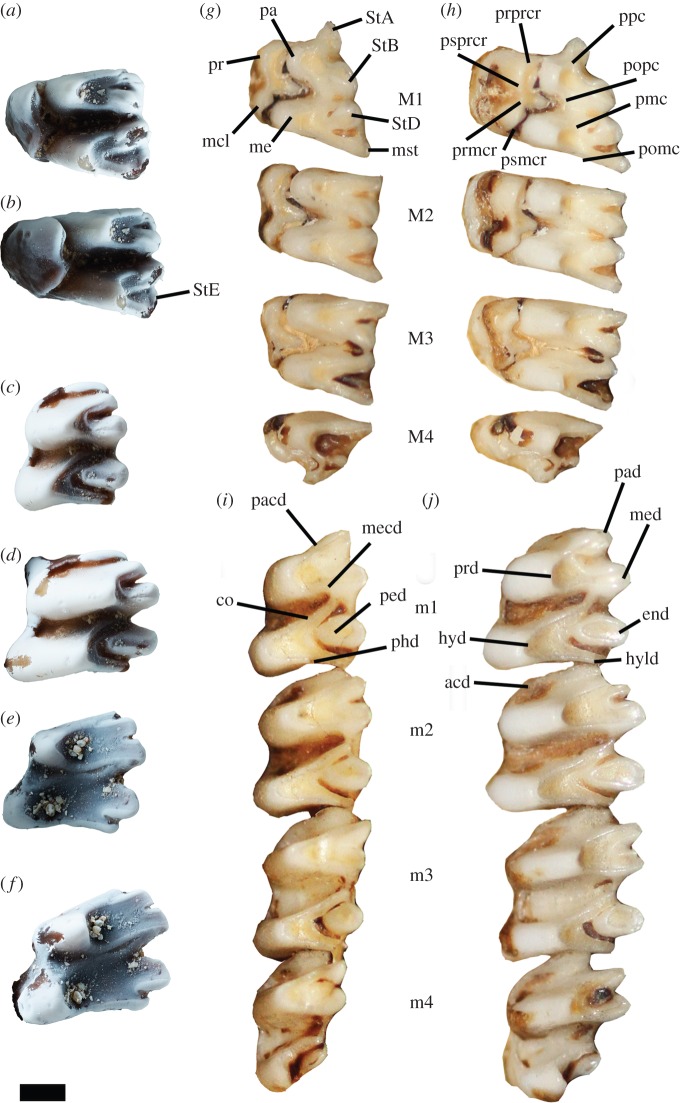
Type specimens of *Chaeropus baynesi* n. sp. (*a*–*f*) compared with modern *C. ecaudatus*, NMV C2900 (*g*–*j*). (*a*,*b*) Holotype NMV P38490, left M3 in occlusal and lingual views; (*c*,*d*) paratype NMV P38492, left m2 in occlusal and buccal views; (*e*,*f*) paratype NMV P38496, worn left m2 in occlusal and buccal views; (*g*,*h*) upper left molars in occlusal and lingual views; (*i*,*j*) lower left molars in occlusal and buccal views. acd, anterior cingulid; end, entoconid; hyd, hypoconid; hyld, hypoconulid; mcl, metaconule; me, metacone; mecd, metacristid; med, metaconid; mst, metastyle; pa, paracone; pacd, paracristid; pad, paraconid; ped, preentocristid; phd, posthypocristid; pmc, premetacrista; pomc, postmetacrista; popc, postparacrista; ppc, preparacrista; pr, protocone; prd, protoconid; prmcr, premetaconule crista; prprcr, preprotocrista; psmcr, postmetaconule crista; psprcr, postprotocrista; StA, StB, StD, StE, stylar cusps A, B, D and E. Scale, 1 mm.

*Holotype.* Left M3 (NMV P38490; figure 2*a*)

*Paratypes.* Left m2 (NMV P38492; figure 2*b*); left worn m2 (NMV P38496; figure 2*c*)

*Type locality and age.* Fisherman's Cliff Local Fauna, Moorna Formation, New South Wales, Australia. It is Late Pliocene to Early Pleistocene in age (2.47–2.92 Ma).

*Diagnosis.* The metaconule of the M3 of *C. baynesi* is less distinct as a cusp than that of *C. ecaudatus,* but the postmetaconule crista ends more posteriorly. The cristid obliqua on the m2 of *C. baynesi* differs from that of *C. ecaudatus* in being less developed (less tall), and ending in the centre of the tooth, instead of the lingual margin of the tooth. Overall, the teeth of *C. baynesi* are smaller in size and less high crowned than *C. ecaudatus*.

*Etymology.* Named in honour of Dr Alexander Baynes for his contribution to the fossil record of *Chaeropus*.

*Description.* The M3 is wider than it is long and distinctively high crowned, but less so compared with *C. ecaudatus* (*Chaeropus* is very odd in the way it increases the height of its crown. It does not increase the height by growing taller cusps, but instead it increases the height by elevating the talon toward the palate, increasing the width of the tooth at an angle. The width measured here is therefore an indication of the height of the crown, with wider teeth meaning increasing hypsodonty. Considering the width of *C. baynesi* is less than that of *C. ecaudatus*, it is therefore less high crowned)*.* StA is worn on the tooth. Both the parastylar and metastylar shelves are short but wide. The postparacrista ends at the base of an oval shaped StB. StD is also oval in shape and connects to the premetacrista lingually. A small StE is present posterior to StD, but no stylar crest is present. The protocone is low on the crown. The preprotocrista ends at the anterolingual base of the paracone. The postprotocrista joins the premetaconule crista posterobuccally. A distinct crest runs buccally from the junction of the postprotocrista and premetaconule crista on the talon between the paracone and metacone, and may be the continuation of the postprotocrista. The metaconule is not distinct and the postmetaconule crista continues to descend the talon posteriorly to end at the lingual base of the metacone. Tooth dimensions are 2.41 mm in length and 3.08 mm in width for NMV P38490.

The m2 is short but wide. The crown is higher on the buccal side than the lingual side, as is typical of *Chaeropus,* but unlike *Isoodon,* the enamel does not cover part of the root. The anterior cingulum is short but wide. The trigonid is short, with the paraconid–metaconid being shorter than the protoconid–paraconid and protoconid–metaconid distances. The talonid is wider than the trigonid, with the hypoconid posterobuccal to the protoconid. Cristid obliqua is poorly developed compared with *C. ecaudatus,* and ends in the centre of the tooth. The posthypocristid is straight and ends at the posterior most corner of the tooth, where the small hypoconulid is located. The entoconid is large, almost triangular in shape, with buccally orientated preentocristid. Tooth dimensions are 2.71 mm in length, 1.69 mm in trigonid width, and 1.89 mm in talonid width for NMV P38492, and 2.75 mm in length, 1.72 mm in trigonid width, and 2.04 mm in talonid width for NMV P38496.

## 3. Discussion

The material of *C. baynesi* described here shows some of the typical synapomorphies for *Chaeropus* [13]*.* The upper and lower molars are high crowned, though less so that in *C. ecaudatus.* The upper molar lacks anterior and posterior cingula as in *C. ecaudatus* but this feature is also found in *Perameles.* In the lower molars of *C. baynesi*, high crown is achieved by the increase in height of the buccal cuspids, as in *C. ecaudatus,* unlike *Isoodon* or *Macrotis,* which achieve high crown by having enamelled roots. Another *Chaeropus* synapomorphy found in *C. baynesi* is the triangular/tear-shaped entoconid (in occlusal view), which is conical in *Perameles, Isoodon* and *Macrotis*, and oval in New Guinea bandicoots (*Peroryctes, Microperoryctes, Echymipera* and *Rhynchomeles*). The only synapomorphy that *C. baynesi* is lacking is the development of the cristid obliqua which is lower and ends further buccal.

The teeth of *C. baynesi* are also slightly smaller than *C. ecaudatus,* suggesting that it was a smaller animal. For example, the length and width of the M3 ranges between 2.8–3.31 mm and 3.82–4.44 m, respectively, in *C. ecaudatus* (measurements from all available specimens registered in the Australian Museum, Museum Victoria and South Australian Museum)*,* significantly larger than the 2.41 mm in length and 3.08 mm in width of *C. baynesi.* The same is true for the m2 of *C. ecaudatus* (length: 2.93–3.54 mm; trigonid width: 2.08–2.67 mm; talonid width: 2.4–2.89 mm) which is also larger than the m2 of *C. baynesi* (length: 2.71–2.75 mm; trigonid width: 1.69–1.72 mm; talonid width: 1.89–2.04 mm). The exact body mass of *C. ecaudatus* is unknown, although it has been estimated at between 200 g [14] and 300 g [4]. *Chaeropus baynesi* is likely to have been below 200 g considering it has smaller teeth than *C.ecaudatus* and therefore a smaller body mass.

Reports of the diet of *C. ecaudatus* have been conflicting, some anecdotes reporting flesh [15] or insects [7], and others grasses [16,17]. Though, analyses of gut contents have showed that it was a highly specialized grazer, with the ability to ferment plant material in its hindgut [4]. Small-bodied grazers are relatively rare, as they have higher mass-specific food requirements without any proportional increase in gut capacity. Minimum size estimates for mammalian grazers is as low as 1 kg [18], or even as low as 600 g for marsupials [19]. With its 200–300 g, *C. ecaudatus* may have been the smallest mammalian grazer to have ever existed. *Chaeropus baynesi,* however, is unlikely to have been a grazer, but more likely to be a mix-feeder or even omnivorous. Not only it is smaller than *C. ecaudatus,* it also has shorter crowns, suggesting that it would wear its teeth down faster under the same diet, and therefore has a shorter life. The poor development of the cristid obliqua on lower molars of *C. baynesi* provides one less blade for shearing of food, one of the important features found in *C. ecaudatus* to be able to reduce grasses to fine particles [4].

*Chaeropus baynesi* is a member of the Fisherman's Cliff Local Fauna in the Moorna Formation, Late Pliocene to Early Pleistocene in age [11]. The local fauna reported, including gastropods, lungfishes, turtles, ratites, dasyurids, bandicoots, wombats, macropods and rodents, lacks arboreal taxa, suggesting that the local environment was rather open [10]. This is the type of environment that *C. ecaudatus* is also known from [14], suggesting that this genus probably evolved in xeric environments. A dated molecular phylogeny of Peramelemorphia has estimated that Chaeropodidae diverged from bandicoots (Peramelidae) and bilbies (Thylacomyidae) sometime in the mid-Late Oligocene [9]. While this estimate is over 20 million years before the oldest fossils for the family presented here, a xeric origin for this family would explain why no such fossils have been recovered, as most of the fossil Peramelemorphian have been recovered from fossil localities in more mesic environments [12,13]. With such a limited fossil record for the family, *C. baynesi* is especially valuable for the understanding of the evolution of this group. With only 2 million years separating *C. baynesi* and *C. ecaudatus,* it is clear that evolution of herbivory in this group was quite rapid, and probably linked climate change during the Pleistocene, though, it is quite extraordinary for such a small mammal to have become a grazer.
