# Correction to ‘Oldest fossil remains of the enigmatic pig-footed bandicoot show rapid herbivorous evolution’

**DOI:** 10.1098/rsos.160778

**Published:** 2016-11-02

**Authors:** Kenny J. Travouillon

**Affiliations:** Western Australian Museum, Welshpool, Western Australia 6106, Australia

## 1. Introduction

This correction is to fulfil the requirements of the International Commission on Zoological Nomenclature (ICZN) code criteria for the publication of new species name. In order for *Chaeropus baynesi* to be a valid taxon, the species name needed to be registered in Zoobank at the time of publication, with the Zoobank number appearing with the publication. This correction aims to solve this issue, and the Zoobank LSIDs numbers are shown below along with a reiteration of the systematic section. The original work should be cited along with the correction when citing the new species.

## 2. Zoobank LSIDs

Original publication (Travouillon [1]):

 urn:lsid:zoobank.org:pub:6E33CED9-3D2D-4E2F-AAB8-C9E6D809C2F2.

This correction:

 urn:lsid:zoobank.org:pub:50459EE4-60C2-4143-99A0-6A7543BF422B.

New species: *Chaeropus baynesi*

 urn:lsid:zoobank.org:act:5EC12F5C-8AB4-412D-A630-31467A4293AD.

## 3. Systematic palaeontology

*Infraclass.* Metatheria.

 *Order.* Peramelemorphia.

 *Family.* Chaeropodidae

 *Genus. Chaeropus*

 *Species. baynesi* new.

 *Holotype.* Left M3 (NMV P38490; figure 2*a*).

 *Paratypes.* Left m2 (NMV P38492; figure 2*b*); left worn m2 (NMV P38496; figure 2*c*).

 *Type locality and age.* Fisherman's Cliff Local Fauna, Moorna Formation, New South Wales, Australia. It is Late Pliocene to Early Pleistocene in age (2.47–2.92 Ma).

 *Diagnosis.* The metaconule of the M3 of *C. baynesi* is less distinct as a cusp than in *C. ecaudatus,* but the postmetaconule crista ends more posteriorly. The cristid obliqua on the m2 of *C. baynesi* differs from that of *C. ecaudatus* in being less developed (less tall), and ending in the centre of the tooth, instead of the lingual margin of the tooth. Overall, the teeth of *C. baynesi* are smaller and less high crowned than *C. ecaudatus*.

 *Etymology.* Named in honour of Dr Alexander Baynes for his contribution to the fossil record of *Chaeropus*.
